# Sensing the quantum limit in scanning tunnelling spectroscopy

**DOI:** 10.1038/ncomms13009

**Published:** 2016-10-06

**Authors:** Christian R. Ast, Berthold Jäck, Jacob Senkpiel, Matthias Eltschka, Markus Etzkorn, Joachim Ankerhold, Klaus Kern

**Affiliations:** 1 Max-Planck-Institut für Festkörperforschung, Heisenbergstraße 1, 70569 Stuttgart, Germany; 2 Institut für Komplexe Quantensysteme and IQST, Universität Ulm, Albert-Einstein-Allee 11, 89069 Ulm, Germany; 3 Institut de Physique, Ecole Polytechnique Fédérale de Lausanne, 1015 Lausanne, Switzerland

## Abstract

The tunnelling current in scanning tunnelling spectroscopy (STS) is typically and often implicitly modelled by a continuous and homogeneous charge flow. If the charging energy of a single-charge quantum sufficiently exceeds the thermal energy, however, the granularity of the current becomes non-negligible. In this quantum limit, the capacitance of the tunnel junction mediates an interaction of the tunnelling electrons with the surrounding electromagnetic environment and becomes a source of noise itself, which cannot be neglected in STS. Using a scanning tunnelling microscope operating at 15 mK, we show that we operate in this quantum limit, which determines the ultimate energy resolution in STS. The *P*(*E*)-theory describes the probability for a tunnelling electron to exchange energy with the environment and can be regarded as the energy resolution function. We experimentally demonstrate this effect with a superconducting aluminium tip and a superconducting aluminium sample, where it is most pronounced.

## Abstract (web summary)

The tunnelling current in scanning tunnelling spectroscopy has often been treated by a continuous charge flow, which lacks proper treatment of charge quantization. Here, Ast *et al*. unveil the effects of granularity in the tunnelling current at extremely low temperatures by including *P*(*E*) theory, thereby reaching the quantum limit in scanning tunnelling spectroscopy.

## Graphical abstract

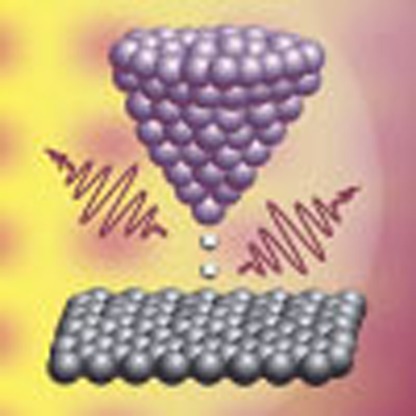

Scanning tunnelling spectroscopy has evolved into one of the most versatile tools to study the electronic structure in real space with atomic precision^1^,^2^,^3^. The differential conductance measured through the tunnelling contact directly accesses the local density of states of the sample. With the growing interest in phenomena with extremely sharp spectral features on smaller and smaller energy scales, the demand for higher and higher spectroscopic energy resolution increases. Examples are the Kondo effect^4^, Yu–Shiba–Rusinov states^5^,^6^, Majorana fermions^7^, or the Josephson effect^8^,^9^,^10^,^11^, just to name a few. Aside from the obvious strategy of lowering the temperature to increase the energy resolution^12^, superconducting tips have successfully been conducted to circumvent the broadening effects of the Fermi function in the tunnelling process and greatly improve the energy resolution^6^,^13^. However, at low temperature *T*, other energy scales such as the charging energy *E*_*C*_ of the tunnel junction may substantially exceed the thermal energy *k*_*B*_*T* such that the granularity of the tunnelling current becomes non-negligible. The question arises whether the tunnelling process encompasses an intrinsic resolution limit, however small it may be, which cannot be overcome. In the following, we will show that the consequences of charge quantization ultimately pose a principal limit to the achievable energy resolution.

The principal properties of a tunnel junction can in many cases be satisfactorily described by a tunnelling resistance^14^ and the charge flow is commonly implicitly assumed to be continuous and homogeneous. However, at very low temperatures, the granularity of the tunnelling current resulting from charge quantization is not necessarily negligible. In this case, the tunnel junction also becomes a capacitor (see Fig. 1a) mediating an interaction of the tunnelling electrons with the surrounding electromagnetic environment^15^,^16^,^17^. This is the quantum limit, where electric charge and magnetic flux (phase) become dual variables (uncertainty principle) so that the charging energy cannot be neglected anymore. As a result, a tunnelling electron may exchange a photon with the surrounding environment, which is accompanied by a loss or gain in energy. This is schematically shown in Fig. 1b for a scanning tunnelling microscope (STM), where the electron tunnels from the tip to the sample and emits a photon to the environment. In addition, the thermal noise of the junction capacitance becomes appreciable for small capacitance values. These effects may be small, but they are non-negligible at low temperatures, especially in the context of ultimate energy resolution.

The so-called *P*(*E*)-theory quantifies the energy exchange with the environment, where the *P*(*E*)-function describes the probability of a tunnelling electron to emit or absorb a photon to or from the environment^16^,^17^,^18^,^19^,^20^. The *P*(*E*)-theory has already been successfully applied in many instances, where tunnelling electrons interact with the surrounding environment, which is modelled as an effective frequency-dependent electromagnetic impedance Z(*ω*). Examples include the Josephson effect in the charge tunnelling regime^18^,^21^, as well as the phenomenon of Coulomb blockade^15^,^16^,^22^,^23^. These examples refer to specific physical situations: tunnelling of Cooper pairs and tunnelling in a high-impedance environment (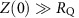, where 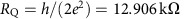 is the resistance quantum), respectively. However, the *P*(*E*)-theory generally applies to inelastic charge transfer processes through tunnel junctions. Accordingly, Pekola *et al*.^24^ have very nicely described a contribution to the superconducting density of states (that is, the Dynes parameter) by environmentally assisted tunnelling through a normalconductor-insulator-superconductor junction. They did not though generalize their findings to the general properties of a tunnel junction.

Here, we show that the photon exchange of tunnelling electrons with the surrounding environment in conjunction with the capacitative junction noise represents a principal limit of the energy resolution Δ*E* in spectroscopic measurements using tunnel junctions (in our case leading to Δ*E* in the low μeV-range). In this regard, the *P*(*E*)-function represents the resolution function of a particular tunnel junction. Using an STM operating at 15 mK (ref. 25), we independently characterize the *P*(*E*)-function of a superconductor-vacuum–superconductor-tunnel junction through its direct relation with the Josephson effect. Subsequently, we demonstrate the impact of the *P*(*E*)-function on the tunnelling process by measuring the superconducting quasiparticle density of states of the same tunnel junction. We find excellent quantitative agreement of our spectra from different tunnel junctions with the model calculations including *P*(*E*)-theory. This leads us to conclude that the *P*(*E*)-function plays a ubiquitous role as a resolution function in the tunnelling process, in particular for energy scales at or below 1 meV.

## Results

### Theoretical modelling

To consider the effects of charge quantization in the tunnelling Hamiltonian, a charge transfer operator has to be included^16^. A detailed derivation can be found in ref. 19. The resulting tunnelling probability 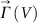 from tip to sample as a function of applied bias voltage is given by refs 16, 26:


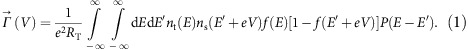


Here, *R*_T_ is the tunnelling resistance, 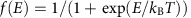 is the Fermi function, and *n*_t_, *n*_s_ are the densities of states of tip and sample, respectively. By exchanging electrons and holes in equation (1), the other tunnelling direction (*V*) from sample to tip can be obtained. Equation (1) differs from the standard expression of the tunnelling probability by the convolution with the *P*(*E*)-function^12^,^27^. If we set *P*(*E*)=*δ*(*E*), which means that there is no energy exchange with the environment and no capacitative noise, the standard expression for the tunnelling current is recovered^28^. It can be clearly seen that the convolution with the *P*(*E*)-function results in a broadening of the spectral features in the density of states. The current *I*(*V*), which is measured through the tunnel junction as a function of applied bias voltage *V*, is the difference of the tunnelling probabilities in the forward 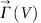 and the backward direction (*V*). The tunnelling current *I*(*V*) is then:


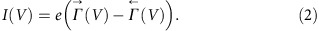


The effect of the capacitance in the tunnel junction and the interaction of the tunnelling electrons with the surrounding environmental impedance has been modelled within the framework of *P*(*E*)-theory, where the *P*(*E*)-function describes the probability for a tunnelling electron to exchange energy with the environment. It is commonly defined through the equilibrium phase correlation function *J*(*t*) as the Fourier transform of exp[*J*(*t*)] (refs 18, 19). To account for the different dissipation channels, we define *J*(*t*)=*J*_0_(*t*)+*J*_N_(*t*), where *J*_0_(*t*) describes the phase correlation of the environmental impedance and *J*_N_(*t*) captures the low frequency capacitative thermal noise in the junction. This allows us to calculate the *P*_0_(*E*)-function for the environmental impedance and the *P*_N_(*E*)-function for the capacitative noise separately. They can be combined to the total *P*(*E*)-function through a convolution (see ‘Methods' section).

Because the correlation function is difficult to calculate directly, we choose an implicit definition for *P*_0_(*E*)^20^:


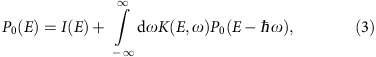


where the two functions *I*(*E*) and *K*(*E*,*ω*) are defined in the ‘Methods' section. The *P*_0_(*E*)-function is parameterized by the temperature *T*, as well as the junction capacitance *C*_J_, and the surrounding impedance Z(*ω*), which together form the total impedance:


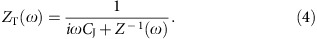


In our STM, the tip acts as a monopole antenna, whose impedance can be modelled in analogy to an infinite transmission line impedance^11^,^18^. The fit parameters are the principal resonance frequency *ω*_0_ and a damping factor *α*. The d.c. resistance *Z*(0) is fixed at the vacuum impedance value of 376.73 Ω. The *P*_N_(*E*)-function is modelled by a normalized Gaussian of width 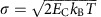 to account for the thermal voltage noise on the junction capacitor. Because both tip and sample are superconducting, we define the charging energy *E*_C_=*Q*^2^/2*C*_J_ using the charge of a Cooper pair (*Q*=2*e*)^18^, which makes it four times higher than the charging energy for a singly charged quasiparticle. Including the capacitative noise has proven essential in previous descriptions of the tunnelling current as well^11^.

### Josephson tunnelling in the dynamical Coulomb blockade regime

The experiments were carried out in an STM operating at a base temperature of 15 mK (ref. 25). We use an Al tip and an Al(100) sample^29^,^30^, which is superconducting at 15 mK (transition temperature *T*_C_=1.1 K). Aluminium has been shown to exhibit minimal intrinsic broadening due to depairing resulting in sharp coherence peaks^31^, which is why it is an excellent material for demonstrating the broadening effects in a tunnel junction, as we will show in the following. We used two different tip wires with diameters of 0.25 and 1 mm (tip 1 and tip 2, respectively) expecting different junction capacitances *C*_J_.

To demonstrate the influence of the *P*(*E*)-function on density of states measurements, we have to independently determine the *P*(*E*)-function from a separate measurement. Since every tip in an STM is slightly different, we have to determine the *P*(*E*)-function for every tip separately. As both tip and sample are superconducting at 15 mK, the most straightforward way to experimentally determine the *P*(*E*)-function is through the Josephson effect. We have to use the *P*(*E*)-theory to describe the Josephson effect rather than the Ivanchenko–Zil'berman (IZ) model, which has been used in STM measurements before, because the IZ model is only valid in the classical phase diffusion limit (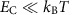)^18^,^32^. In the case of a simple ohmic impedance (and only then), a capacitative element renormalizes the Josephson energy, so that the IZ model can still be used^33^,^34^. This is clearly not the case here. Furthermore, as has been shown before, the environmental impedance in our experiment exhibits resonances and cannot be approximated by the generic impedance in the IZ model^11^,^35^. In the sequential charge tunnelling regime, where the charging energy *E*_C_ is larger than the Josephson energy *E*_J_ (which is commonly the case in a standard STM setup), the current–voltage characteristics is given by ref. 18:


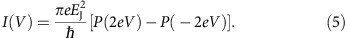


The Josephson energy *E*_J_, can be regarded as a scaling parameter here^36^. In this sense, the *I*(*V*) measurement of the Josephson effect is a direct measure of the *P*(*E*)-function^18^,^19^.

**Figure 2:**
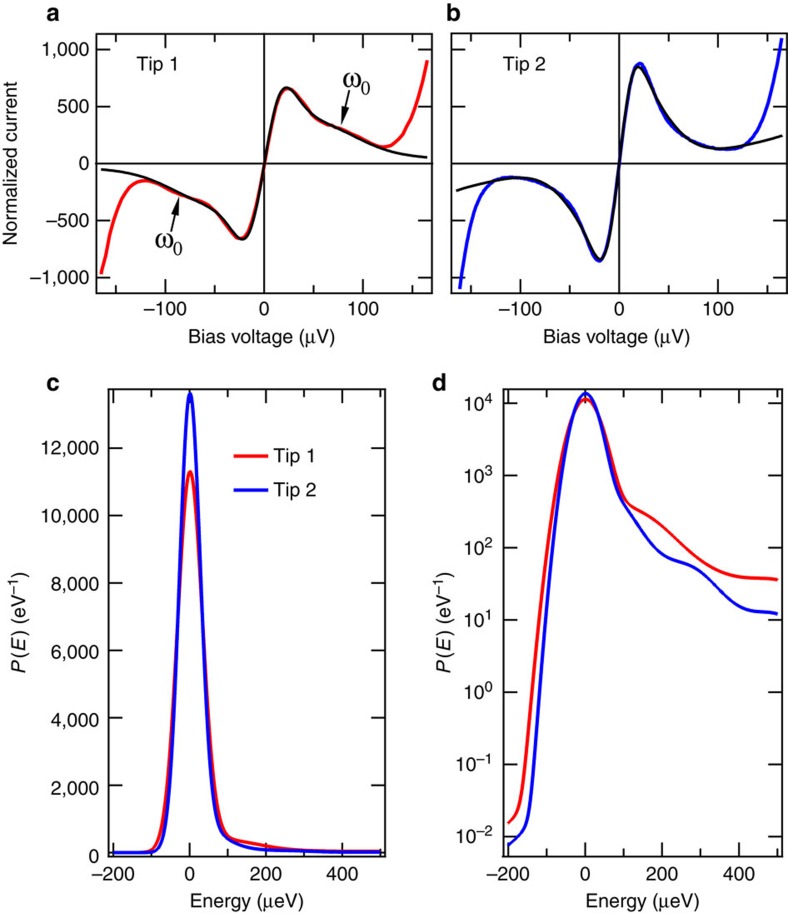
Josephson current–voltage characteristics. *I*(*V*)-characteristics of the Josephson effect for two different Al tips on an Al(100) sample. The tunnel junctions in (**a**) and (**b**) have a capacitance of 3.5 fF and 7 fF, respectively, which means that the tunnel junction in (**a**) is more sensitive to the surrounding environment. Therefore, the principal impedance resonance *ω*_0_ is visible in (**a**) and not in (**b**). The fit using *P*(*E*)-theory (black lines) is in excellent agreement with the data. The *P*(*E*)-functions extracted from the fits in (**a**) and (**b**) are shown on a linear scale in (**c**) and on a logarithmic scale in (**d**). The asymmetry of the *P*(*E*)-function is clearly visible.

We have measured the *I*(*V*)-characteristics of the Josephson effect for two different aluminium tips, which are shown in Fig. 2. The tunnelling conditions were such that for both tips the current setpoint was 5 nA at a voltage of 1 and 2 meV for tip 1 and 2, respectively. For better comparison, the current was divided by 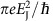. The general features of the Josephson effect in the sequential charge tunnelling regime are visible, however, the peak in Fig. 2b is somewhat higher and sharper than in panel (a). In addition, the spectrum in panel (a) shows a broad peak of the principal antenna resonance *ω*_0_. The fits using equation (5) are shown as black lines (see Supplementary Note 1). They agree well with the measured data. The most significant difference between the two tunnel junctions is that the junction in the Fig. 2a has a capacitance of *C*_J_=3.5±0.2 fF, while in Fig. 2b the capacitance is *C*_J_=7.0±0.1 fF, which can be traced back to the different wire diameters (see ‘Methods' section). The lower the junction capacitance value *C*_J_ is, the more sensitive the junction will be to the environment *Z*(*ω*) (cf. equation (8)).

The actual shape of the *P*(*E*)-function for the two tips is shown in Fig. 2c and on a semi-log scale in (d). The full-width at half maximum (FWHM) for tip 1 and 2 is 77.2 μeV and 65.4 μeV, respectively, which will have a non-negligible effect on the superconducting density of states. In addition, the *P*_0_(*E*)-function obeys the detailed balance symmetry^20^, 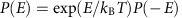, which makes the function inherently asymmetric as can be clearly seen in Fig. 2d even after the convolution with the capacitative noise. With the well-defined *P*(*E*)-function, we can look at its impact on the details of a quasiparticle spectrum.

### Charge quantization effects in quasiparticle spectra

**Figure 3:**
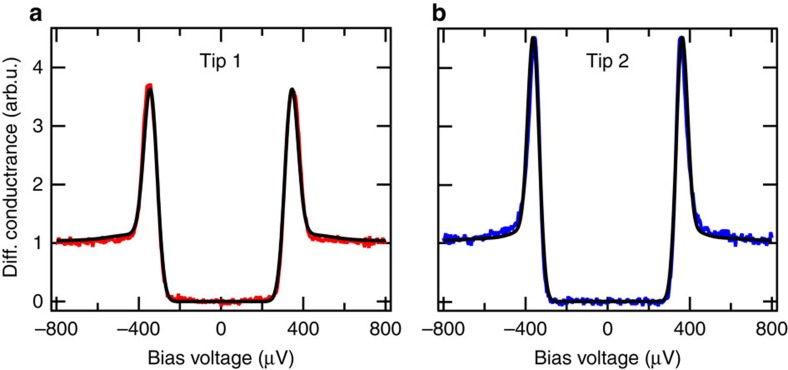
Quasiparticle spectra. Differential conductance spectra of the superconducting densities of states for the two different Al tips on an Al(100) sample (the tip wire diameters are 0.25mm in (**a**) and 1mm in (**b**)). To suppress subgap features, we have measured at low transmission (stabilization at 2 meV and 50 pA). The fits are shown as black lines with excellent agreement. The superconducting density of states of both tip and sample was modeled by the simple Bardee–Cooper–Schrieffer (BCS)-model without any additional broadening parameters.

The differential conductance d*I*/d*V* spectra measured with the two tips as a function of bias voltage *V* are shown in Fig. 3. The lock-in modulation for the spectra was 10 μeV (peak-to-peak) at 724 Hz. Because both the tip and the sample are superconducting, the apparent gap in the spectrum has a width of 2(*Δ*_t_+*Δ*_s_). The tip gap *Δ*_t_ can be slightly smaller than the bulk value of 180 μeV. The most noticeable difference between the two spectra is the height of the coherence peaks. We fit the two spectra with the differential conductance model obtained from the derivative of equation (2) in combination with equation (1). For the superconducting density of states in tip *n*_t_ and sample *n*_s_, we use the simple BCS model^37^, explicitly neglecting any intrinsic broadening (for example, Dynes parameter *Γ* (ref. 38)):


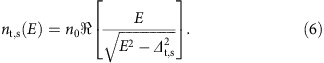


For the *P*(*E*)-function we use the same values that have been obtained from the fit to the data in Fig. 2, which means that the number of fit parameters is reduced to the value of the superconducting gap *Δ* and an overall scaling factor including the tunnelling resistance *R*_T_. We find excellent agreement for both spectra using the corresponding *P*(*E*)-function and with gap values of *Δ*_t_=160±2 μeV for tip 1 and *Δ*_t_=180±2 μeV for tip 2. The sample gap is set to the bulk value *Δ*_s_=180 μeV. In both cases, the height and the shape of the coherence peaks are quantitatively well reproduced.

By contrast, disregarding the *P*(*E*)-function and fitting the spectra in Fig. 3 with the Dynes equation^38^ to account for the broadening, does not give a satisfactory fit at all (see Supplementary Note 2 and Supplementary Figure 1). The height reduction in the coherence peaks has to be absorbed into the empirical broadening parameter *Γ*. This leads to the accumulation of quasiparticle spectral weight inside the gap, which is not observed experimentally. In fact, as outlined in the ‘Methods' section, the Coulomb dip found in the normal conducting state of tip and sample strongly supports our *P*(*E*)-analysis. Since it is well described by the same *P*(*E*)-function as for the quasiparticle spectrum and the Josephson spectrum, we attribute the reduction of the singularities to sharp peaks to the *P*(*E*)-function broadening.

## Discussion

According to the *P*(*E*)-theory, the exchange of energy with the environment during the tunnelling process should be an ubiquitous phenomenon^16^. In the majority of cases, it has been discussed in the context of dynamical Coulomb blockade (DCB)^15^,^16^,^22^,^23^ as well as the Josephson effect in the sequential charge tunnelling regime^17^,^18^,^21^. While in these cases, the role of the *P*(*E*)-theory is obvious, its role as a resolution function in every tunnelling spectrum is more subtle, but non-negligible, as we will show in the following. Neglecting the asymmetric shape for a moment and looking at the general broadening effect of the *P*(*E*)-function, we find that the FWHM of the *P*(*E*)-function is dominated by the capacitative noise and is essentially determined by the junction capacitance *C*_J_ as well as the temperature *T* (see also Supplementary Note 3).

**Figure 4:**
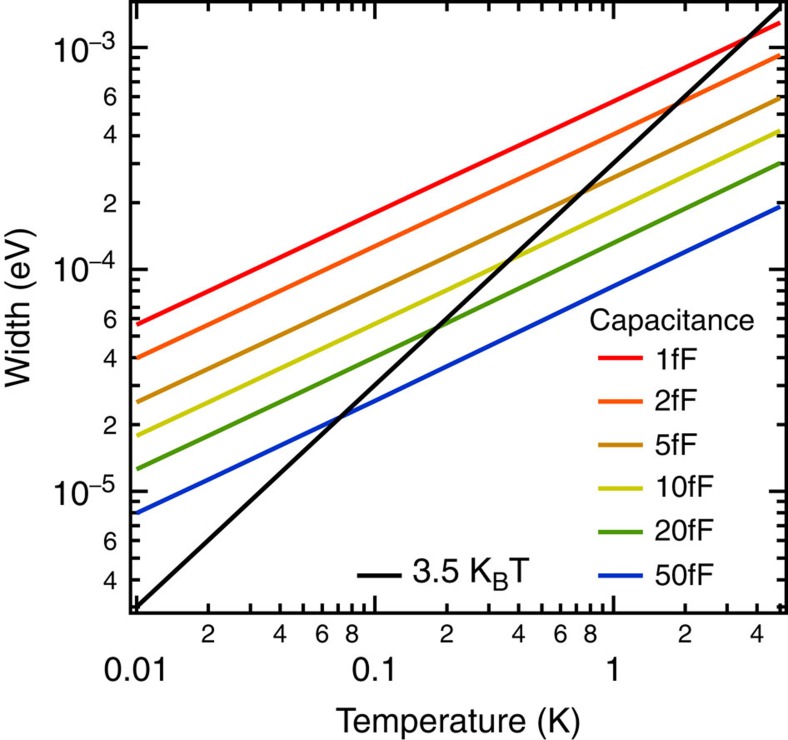
Energy broadening contributions. Energy broadening due to *P*(*E*)-broadening from the environment in comparison to thermal broadening from the Fermi functions as a function of temperature. The *P*(*E*)-broadening is shown for typical junction capacitances that can be found in an STM tunnel junction (see ‘Methods' Section). For the total energy broadening and hence the resolution limit both contributions have to be combined.

The FWHM of the *P*(*E*)-function for typical low temperatures (0.01–5 K) and capacitances (1–50 fF) is shown in Fig. 4. For comparison, the conventional thermal broadening Δ*E*_therm_ of differential conductance spectra in the STM due to the Fermi function is also shown as a black line (Δ*E*_therm_=3.5 *k*_B_*T*). While the thermal Fermi function broadening depends linearly on temperature, we find an overall empirical relation for the effective energy resolution due to *P*(*E*)-broadening, which is a function of temperature and capacitance 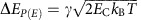. The coefficient *γ* has an average value of *γ*=2.45±0.1, keeping in mind that the capacitative noise is the dominant contribution to the FWHM. The *P*_0_(*E*)-function changes the coefficient *γ* slightly depending on the actual values of the parameters. This means that for low-enough temperature, the *P*(*E*)-broadening will eventually be the dominant contribution to the resolution limit, regardless of whether the tip and/or sample are superconducting or not. We note that this empirical equation holds for capacitive noise from Cooper pairs. For noise from quasiparticles, we expect the *P*(*E*)-broadening to be reduced by about one half. At or below 1 K, the *P*(*E*)-broadening definitely has to be taken into account when optimizing the energy resolution. The optimizing strategy will be to increase the junction capacitance by appropriate *ex situ* tip shaping on a macroscopic scale (up to mm-scale). Increasing the junction capacitance will increase the crosstalk between tip and sample, so that a trade-off between energy resolution and STM performance will have to be made.

Due to the asymmetry of the *P*(*E*)-function, the spectral features in the density of states will not only be broadened, but may also change shape, which can have a strong influence on the interpretation of experimental data. The asymmetry evens out for higher temperatures, but at low temperatures, it has to be considered as can be seen in the fits of the differential conductance spectra in Fig. 3. If a symmetric broadening had been sufficient to fit these spectra, a Dynes fit would likely have sufficed. We expect the *P*(*E*)-broadening to be most significant on intrinsically sharp spectral features, such as coherence peaks of a superconducting gap. In addition, sharp Kondo peaks with a low Kondo temperature on the order of 1 K may show an effectively higher Kondo temperature when the *P*(*E*)-broadening is not taken into account. Also, Yu–Shiba–Rusinov states, which have an intrinsically *δ*-like spectral appearance^39^, will be strongly influenced by *P*(*E*)-broadening.

In summary, we have shown that the interaction of tunnelling electrons with the environmental impedance as well as the capacitative junction noise limit the effective energy resolution in spectroscopic measurements of the differential conductance with the STM. The *P*(*E*)-function that models the energy exchange with the electromagnetic environment combined with the capacitative noise is the energy resolution function of the tunnel junction. The effect of *P*(*E*)-broadening becomes dominant at or below 1 K and has to be taken into account when optimizing the energy resolution. In this regime, the quantum nature of the tunnelling process becomes evident, there is virtually no elastic tunnelling in experiments, and the surrounding electromagnetic environment has to be taken into account.

## Methods

### Sample preparation

The sample that was used was an Al(100) single crystal and the tip was an Al wire of 99.9999% purity. The sample was sputtered (Ar^+^ ions at 500 eV) and annealed in ultrahigh vacuum (low 10^−10^ mbar range) in several cycles, while the tip was cut in air, transferred in vacuum and then sputtered (Ar^+^ ions at 500 eV) to remove the native oxide. With a superconducting transition temperature *T*_C_=1.1 K both tip and sample are superconducting at 15 mK with a fully open gap. The quasiparticle density of states of aluminium in the superconducting state has a very Bardeen–Cooper–Schrieffer (BCS)-like character with minimal intrinsic broadening^31^, which is why aluminium is an excellent material for demonstrating the broadening effects in a tunnel junction.

### The dynamical Coulomb blockade regime

**Figure 5:**
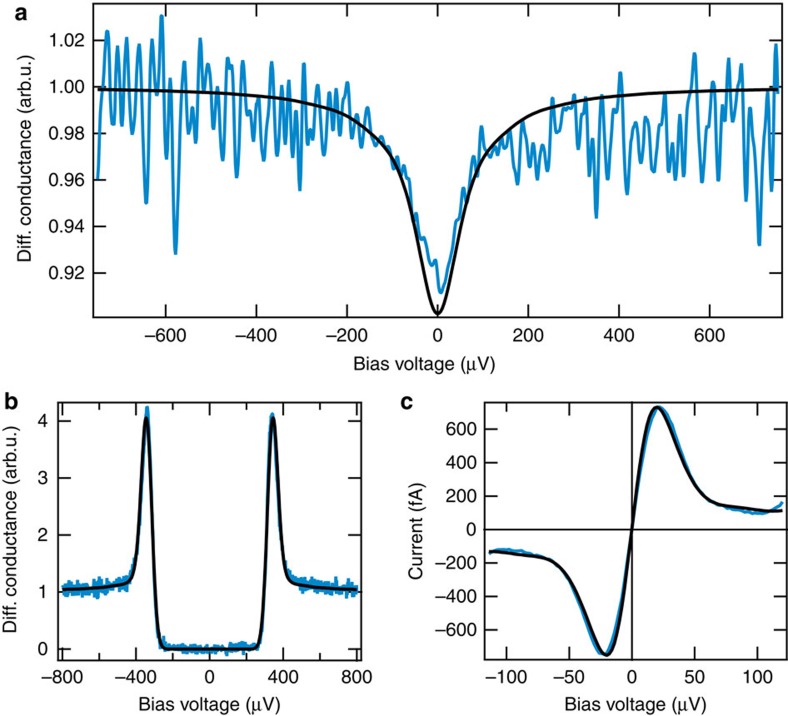
Dynamical Coulomb blockade. (**a**) Differential conductance spectrum in the normal state after applying a 10 mT magnetic field to quench superconductivity in the tip and the sample. The conductance dip at zero bias clearly indicates a DCB as a result of charge quantization effects. The quasiparticle spectrum of the superconducting Al tip and sample in (**b**) and the Josephson spectrum in (**c**) were fitted with the same *P*(*E*)-function as used in (**a**).

We assume a sequential tunnelling regime, where the system, including the environment, is relaxed before the next quasiparticle or Cooper pair tunnels. To support our treatment of inelastic Cooper pair/quasiparticle tunnelling in the main text, we here show data for the normal conducting regime. Accordingly, DCB should then induce a characteristic dip in the differential conductance around zero voltage. This is indeed found in Fig. 5a, which shows the differential conductance spectrum taken with the Al tip and the Al sample in the normal conducting state in a 10 mT magnetic field. The spectrum clearly shows a conductance dip by about 8% demonstrating a DCB in our system. This dip is clear evidence that the consequences of charge quantization cannot be neglected in our operating regime. Consequently, we have to take the effects of the tunnelling capacitance into account and conduct *P*(*E*)-theory to describe our data^21^. The spectra in Fig. 5b,c show the quasiparticle spectrum in the superconducting state (at 0 mT) and the Josephson effect, respectively. Both of these spectra were fitted with the same *P*(*E*)-function as in Fig. 5a having a capacitance *C*_J_=7 fF (tip wire with 1 mm diameter), a resonance energy 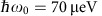 and a damping factor *α*=0.75 (the details of these parameters are described below). The fitted temperature was 100 mK. For the spectrum in Fig. 5a, we used the charging energy *E*_C_ for quasiparticles, because both tip and sample are in the normal conducting state. For the spectrum in Fig. 5b,c, we used the charging energy for Cooper pairs.

### Modelling the superconducting gap

We model the superconducting gap of aluminium with the simple BCS model because we work with high purity tip and sample material, so that we assume that there is negligible depairing due to impurities in the material. Further, the tip is solid aluminium wire, and we have not observed any indications of confinement effects. As such, there is *a priori* no real justification to describe the superconducting gap with Maki's equation or the Usadel equation^40^,^41^, although the functions may give a satisfactory fit. Conducting *P*(*E*)-theory gives an overall consistent picture and describes the superconduting quaisparticle spectrum with the simplest BCS model demonstrating that *P*(*E*)-broadening is the dominating broadening mechanism in our case.

### Modelling the environmental impedance

In our STM the surrounding impedance that contributes to the *P*(*E*)-function is the vacuum as well as the tip acting as a monopole antenna with a corresponding resonance spectrum that depends on the length of the tip^11^. Approximating the resonance spectrum by an infinite transmission line impedance^11^,^18^, we find an analytic expression for the impedance *Z*(*ω*):


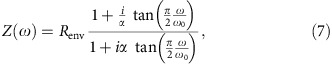


where *R*_env_ is the effective d.c. resistance of the environmental impedance, *α* is an effective damping parameter and *ω*_0_ is the frequency of the principal resonance. The parameter *R*_env_ is set to the vacuum impedance of 376.73 Ω. The fit parameters for this impedance are *α* and *ω*_0_.

The total impedance *Z*_T_(*ω*) takes into account the capacitance *C*_J_ in the tunnel junction as well:


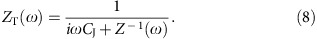


Here, the parameter *C*_J_ is also a fit parameter.

### Calculating the *P(E)*-function

The *P*(*E*)-function is commonly defined through the equilibrium phase correlation function *J*(*t*) through^18^:


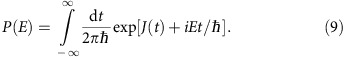


We regard the energy exchange with the environmental impedance and the capacitative noise from the tunnel junction as two independent processes, which allows us to separate the correlation function as:


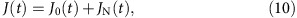


where *J*_0_(*t*) is the phase correlation function from the environmental impedance and *J*_N_(*t*) is due to the capacitative junction noise. We can then calculate the corresponding probability functions separately, where *P*_0_(*E*) is the probability due to the interaction with the environmental impedance and *P*_N_(*E*) is due to the capacitative noise. Exploiting the convolution theorem, we can calculate the total *P*(*E*)-function through a convolution:


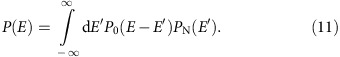


For the calculation of the *P*_0_(*E*)-function for the environmental impedance, we follow the implementation given in ref. 20. The *P*_0_(*E*)-function is calculated through an indirect definition within an integral equation:


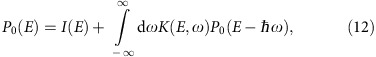


where *K*(*E*,*ω*) is the integral kernel. The inhomogeneity *I*(*E*) is defined as:


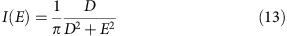


with


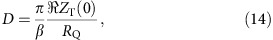


where 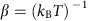, *T* is the temperature, and *R*_Q_=*h*/(2*e*^2^) is the resistance quantum. The integral kernel *K*(*E*,*ω*) is defined as:


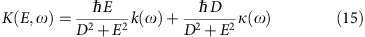


with the functions *k*(*ω*) and *κ*(*ω*) being:


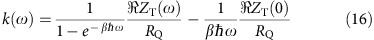



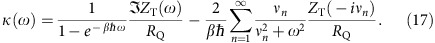


The Matsubara frequencies *v*_*n*_ are defined as 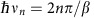. Using the inhomogeneity *I*(*E*) as a starting value for the *P*_0_(*E*)-function calculation, the integral equation in equation (12) can be solved self-consistently. Convergence is usually reached within a few iterations. Treating the integral as a convolution, the calculation can be done very efficiently numerically. Care should be taken to extend the integral range to sufficiently large energies, while at the same time having a high enough numerical point density. Other than the impedance *Z*_T_(*ω*), the temperature *T* is a fit parameter in this part of the *P*(*E*)-function calculation.

The *P*_N_(*E*)-function for the thermal capacitative noise of the tunnel junction has proven a non-negligible part of the total *P*(*E*)-function. The low frequency capacitative noise *P*_N_(*E*) is modelled by a Gaussian^18^:


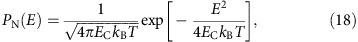


where *E*_C_=*Q*^2^/2*C*_J_ is the charging energy for Cooper pairs (*Q*=2*e*). The *P*_N_(*E*)-function does not introduce any new fit parameters as the junction capacitance *C*_J_ as well as the temperature *T* are already defined in the *P*_0_(*E*)-function.

### Estimating the junction capacitance

**Figure 6:**
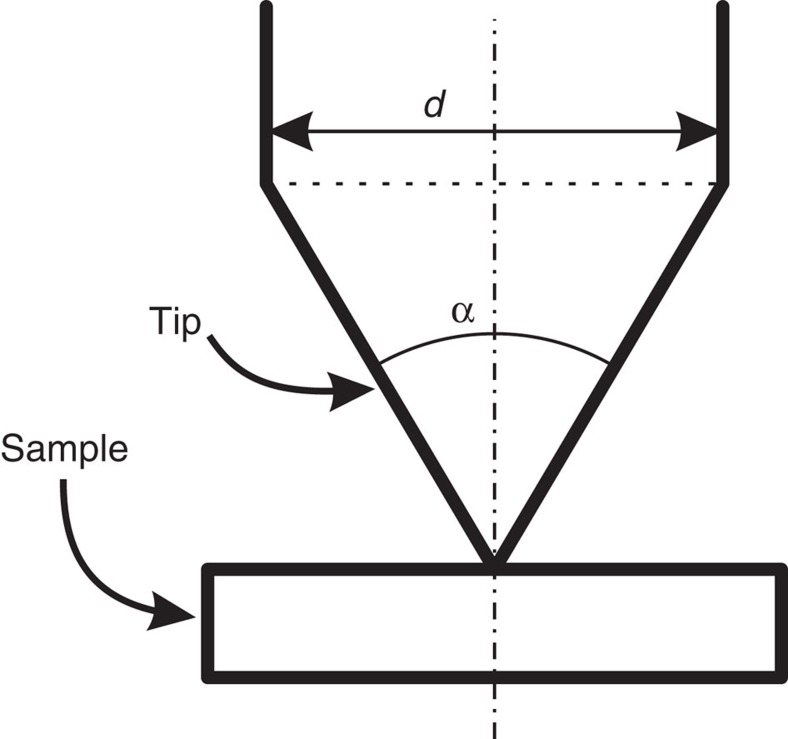
Capacitance model for the tunnel junction. Simple model of tip and sample for estimating the junction capacitance. The tip is assumed to be conical and touches the (flat) sample in one point. The diameter *d* of the tip wire as well as the opening angle *α* are indicated.

The capacitance of the tunnel junction cannot be easily calculated as the details of the tip shape of the apex are unknown. However, it is possible to estimate the contributions to the junction capacitance from a simple model that can be calculated analytically. Assuming a conical tip that touches a flat sample in one point (see Fig. 6), we can calculate the junction capacitance *C*_J_ using spherical coordinates:


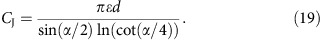


This model shows that for the junction capacitance the full diameter *d* of the tip has to be considered. For a tip wire diameter *d* of 0.5 mm and an opening angle *α* of 60°, we calculate a junction capacitance of about 21 fF. In the experiment we have used wire diameters of 0.25 mm (tip 1) and 1 mm (tip 2), with junction capacitances of 3.5 and 7 fF, respectively. Considering the crudeness of the model, we find good agreement between experiment and calculation.

From the total impedance *Z*_T_(*ω*) in equation (8), we find that the larger the junction capacitance *C*_J_, the less sensitive the junction will be to the environment, the smaller the capacitative noise will be, and, correspondingly, the better the energy resolution will be. Thus, the diameter of the tip plays a crucial role. The length of the tip will essentially determine the position of the antenna resonances, which is less crucial to the energy resolution.

### Data availability

The authors declare that the data supporting the findings of this study are available within the article and its supplementary information.

## Additional information

**How to cite this article:** Ast, C. R. *et al*. Sensing the quantum limit in scanning tunnelling spectroscopy. *Nat. Commun.* **7,** 13009 doi: 10.1038/ncomms13009 (2016).

## Supplementary Material

Supplementary Information Supplementary Figures 1, Supplementary Notes 1-3 and Supplementary References.

## Figures and Tables

**Figure 1:**
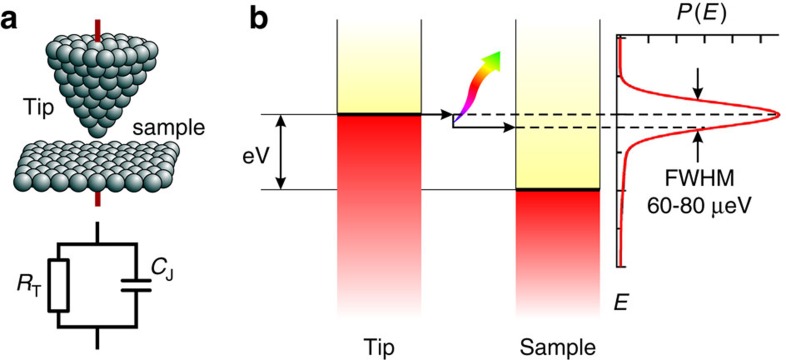
Schematic of the tunnelling process. (**a**) Schematic drawing of an STM tunnel junction consisting of tip and sample. The equivalent circuit diagram is represented by a tunnelling resistor *R*_T_ and the junction capacitance *C*_J_. (**b**) Schematic energy diagram showing the energy loss of an electron tunnelling in an STM from the tip to the sample. Interacting with the surrounding environment, the electron loses energy according to the probability given by the *P*(*E*)-function. The FWHM is indicated with the values found in this work.

## References

[b1] Wiesendanger R. Spin mapping at the nanoscale and atomic scale. Rev. Mod. Phys. 81, 1495 (2009).

[b2] Bode M. Spin-polarized scanning tunnelling microscopy. Rep. Prog. Phys. 66, 523–582 (2003).

[b3] Leemput L. E. C. v. d. & Kempen H. v. Scanning tunnelling microscopy. Rep. Prog. Phys. 55, 1165 (1992).

[b4] Zhang Y.-h. . Temperature and magnetic field dependence of a Kondo system in the weak coupling regime. Nat. Commun. 4, 2110 (2013). 23817525 10.1038/ncomms3110 PMC3730050

[b5] Yazdani A., Jones B. A., Lutz C. P., Crommie M. F. & Eigler D. M. Probing the local effects of magnetic impurities on superconductivity. Science 275, 1767–1770 (1997). 9065395 10.1126/science.275.5307.1767

[b6] Franke K. J., Schulze G. & Pascual J. I. Competition of superconducting phenomena and kondo screening at the nanoscale. Science 332, 940–944 (2011). 21596987 10.1126/science.1202204

[b7] Nadj-Perge S. . Observation of Majorana fermions in ferromagnetic atomic chains on a superconductor. Science 346, 602–607 (2014). 25278507 10.1126/science.1259327

[b8] Naaman O., Teizer W. & Dynes R. C. Fluctuation dominated Josephson tunneling with a scanning tunneling microscope. Phys. Rev. Lett. 87, 097004 (2001). 11531592 10.1103/PhysRevLett.87.097004

[b9] Kimura H., Barber R. P., Ono S., Ando Y. & Dynes R. C. Josephson scanning tunneling microscopy: a local and direct probe of the superconducting order parameter. Phys. Rev. B 80, 144506 (2009).

[b10] Rodrigo J. G., Crespo V. & Vieira S. Josephson current at atomic scale: tunneling and nanocontacts using a STM. Phys. C 437–438, 270–273 (2006).

[b11] Jäck B. . A nanoscale gigahertz source realized with Josephson scanning tunneling microscopy. Appl. Phys. Lett. 106, 013109 (2015).

[b12] Tersoff J. & Hamann D. R. Theory of the scanning tunneling microscope. Phys. Rev. B 31, 805–813 (1985). 10.1103/physrevb.31.805 9935822

[b13] Pan S. H., Hudson E. W. & Davis J. C. Vacuum tunneling of superconducting quasiparticles from atomically sharp scanning tunneling microscope tips. Appl. Phys. Lett. 73, 2992–2994 (1998).

[b14] Chen C. J. Introduction To Scanning Tunneling Microscopy Oxford Univ. Press (2008).

[b15] Delsing P., Likharev K. K., Kuzmin L. S. & Claeson T. Effect of high-frequency electrodynamic environment on the single-electron tunneling in ultrasmall junctions. Phys. Rev. Lett. 63, 1180–1183 (1989). 10040490 10.1103/PhysRevLett.63.1180

[b16] Devoret M. H. . Effect of the electromagnetic environment on the coulomb blockade in ultrasmall tunnel junctions. Phys. Rev. Lett. 64, 1824 (1990). 10041498 10.1103/PhysRevLett.64.1824

[b17] Averin D., Nazarov Y. & Odintsov A. Incoherent tunneling of the cooper pairs and magnetic flux quanta in ultrasmall Josephson junctions. Phys. B 165–166, 945 (1990).

[b18] Ingold G., Grabert H. & Eberhardt U. Cooper-pair current through ultrasmall Josephson junctions. Phys. Rev. B 50, 395 (1994). 10.1103/physrevb.50.395 9974556

[b19] Ingold G.-L. & Nazarov Y. V. Single Charge Tunneling, chap. Charge Tunneling Rates in Ultrasmall Junctions Plenum Press (1992).

[b20] Ingold G.-L. & Grabert H. Finite-temperature current-voltage characteristics of ultrasmall tunnel junctions. Europhys. Lett. 14, 371 (1991).

[b21] Hofheinz M. . Bright side of the Coulomb blockade. Phys. Rev. Lett. 106, 217005 (2011). 21699333 10.1103/PhysRevLett.106.217005

[b22] Brun C. . Dynamical Coulomb blockade observed in nanosized electrical contacts. Phys. Rev. Lett. 108, 126802 (2012). 22540609 10.1103/PhysRevLett.108.126802

[b23] Serrier-Garcia L. . Scanning tunneling spectroscopy study of the proximity effect in a disordered two-dimensional metal. Phys. Rev. Lett. 110, 157003 (2013). 25167301 10.1103/PhysRevLett.110.157003

[b24] Pekola J. P. . Environment-assisted tunneling as an origin of the dynes density of states. Phys. Rev. Lett. 105, 026803 (2010). 20867725 10.1103/PhysRevLett.105.026803

[b25] Assig M. . A 10 mK scanning tunneling microscope operating in ultra high vacuum and high magnetic fields. Rev. Sci. Inst 84, 033903 (2013). 10.1063/1.4793793 23556826

[b26] Odintsov A. Effect of dissipation on the characteristics of small-area tunnel junctions: application of the polaron model. Sov. Phys. JETP 67, 1265 (1988) (Russian original - *ZhETF*, **94**, 312, 1988)).

[b27] Bardeen J. Tunnelling from a many-particle point of view. Phys. Rev. Lett. 6, 57–59 (1961).

[b28] Chen C. J. Theory of scanning tunneling spectroscopy. J. Vac. Sci. Technol 6, 319–322 (1988).

[b29] Guillamon I., Suderow H., Vieira S. & Rodiere P. Scanning tunneling spectroscopy with superconducting tips of Al. Phys. C 468, 537–542 (2008).

[b30] Rodrigo J. G., Suderow H. & Vieira S. On the use of STM superconducting tips at very low temperatures. Eur. Phys. J. B 40, 483–488 (2004).

[b31] Giaever I., Hart H. R. & Megerle K. Tunneling into superconductors at temperatures below 1°K. Phys. Rev. 126, 941–948 (1962).

[b32] Ivanchenko Y. M. & Zil'berman L. A. The Josephson effect in small tunnel contacts. Sov. Phys. JETP 28, 1272 (1969).

[b33] Grabert H., Ingold G.-L. & Paul B. Phase diffusion and charging effects in Josephson junctions. Europhys. Lett. 44, 360–366 (1998).

[b34] Ankerhold J. . Overdamped quantum phase diffusion and charging effects in Josephson junctions. Europhys. Lett. 67, 280 (2004).

[b35] Jäck B. . Critical Josephson current in the dynamical Coulomb blockade regime. Phys. Rev. B 93, 020504 (2016).

[b36] Ambegaokar V. & Baratoff A. Tunneling between superconductors. Phys. Rev. Lett. 10, 486–489 (1963).

[b37] Bardeen J., Cooper L. N. & Schrieffer J. R. Theory of superconductivity. Phys. Rev. 108, 1175–1204 (1957).

[b38] Dynes R. C., Narayanamurti V. & Garno J. P. Direct measurement of quasiparticle-lifetime broadening in a strong-coupled superconductor. Phys. Rev. Lett. 41, 1509–1512 (1978).

[b39] Salkola M. I., Balatsky A. V. & Schrieffer J. R. Spectral properties of quasiparticle excitations induced by magnetic moments in superconductors. Phys. Rev. B 55, 12648–12661 (1997).

[b40] Worledge D. & Geballe T. Maki analysis of spin-polarized tunneling in an oxide ferromagnet. Phys. Rev. B 62, 447–451 (2000).

[b41] Usadel K. D. Generalized diffusion equation for superconducting alloys. Phys. Rev. Lett. 25, 507–509 (1970).

