# Supplementary material for: Sensing the quantum limit in scanning tunnelling spectroscopy
Source: Nat Commun. 2016 Oct 6;7:13009. doi: 10.1038/ncomms13009 (PMC5059741; doi:10.1038/ncomms13009)
Supplement: Supplementary Information — Supplementary Figures 1, Supplementary Notes 1-3 and Supplementary References. [file ncomms13009-s1.pdf]

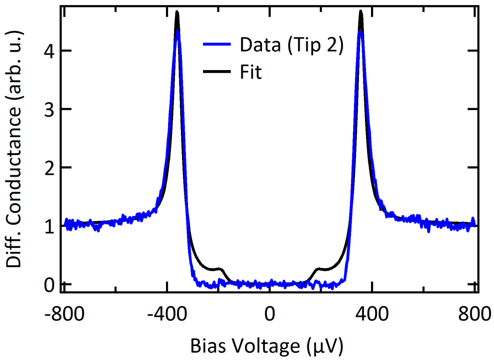

Supplementary Figure 1: **Quasiparticle spectrum with Dynes fit.** Differential conductance spectrum of tip 2 (blue) fitted by a Dynes function (black) disregarding the $P(E)$-broadening. The reduction of the singularity in the coherence peaks to finite values results in high Γ-parameters, which also fill the gap resulting in an unrealistic fit.

SUPPLEMENTARY NOTE 1: FIT PARAMETERS

In the present work, we find $\hbar\omega_0 = 233 \pm 10\,\mu$eV, $\alpha = 0.7 \pm 0.02$, and $C_J = 3.5 \pm 0.2$ fF for tip 1 as well as $\hbar\omega_0 = 120 \pm 15\,\mu$eV, $\alpha = 0.75 \pm 0.05$, and $C_J = 7.0 \pm 0.1$ fF for tip 2. The fitted temperatures were 65 ± 5 mK and 92 ± 2 mK for tip 1 and 2, respectively. They agree well with the corresponding parameters in Ref. [1]. Further, the values for the critical current I_C used to fit the Josephson curves were 1.6±0.1 nA and 0.66±0.05 nA for tip 1 and tip 2, respectively. From the tunneling resistance and the values of the gaps, we can calculate the expected critical current from the Ambegaokar-Baratoff formula to 1.46 nA and 0.71 nA for tip 1 and tip 2, respectively [2]. Both values lie within 10% of the fit values, which gives an additional, independent consistency check to the overall suitability of the model that we are using.

SUPPLEMENTARY NOTE 2: DYNES FIT

In Supplementary Fig. 1, the differential conductance spectrum of tip 2 has been fitted with the Dynes equation [3]:

$$n_{t,s}(E) = n_0 \Re \left[\frac{E + i\Gamma}{\sqrt{(E + i\Gamma)^2 - \Delta_{t,s}^2}} \right], \tag{1}$$

where the phenomenological parameter Γ introduces a general broadening of the BCS density of states. The fit neglects the $P(E)$-broadening, which means that the reduction of the singularities to finite values has to be absorbed in the Γ-parameter. Consequently, the broadening results in a rather large value $\Gamma = 10.8 \pm 0.5\,\mu$eV. At the same time, there is a sizeable filling of the gap, which makes the Dynes model unsuitable for fitting these spectra. In this sense, we attribute the much better fit using the $P(E)$-function broadening also to the inherent asymmetry of the $P(E)$-function strengthening the validity of our approach.

SUPPLEMENTARY NOTE 3: DIFFERENT BROADENING MECHANISMS

Different contributions to the effective broadening can be identified: thermal broadening, broadening due to the AC modulation of the lock-in amplifier, high-frequency (RF) noise, as well as the broadening due to the $P(E)$-function, which are discussed and compared in the following:

Thermal broadening. Just for an estimate we take the best temperature value of 15 mK (thermometer reading) and the worst temperature value (fit value) not knowing what the electron temperature in the tip and the sample really are. The thermal energy $k_B T$ ranges from 1.3 μeV to 7.7 μeV. At these temperatures the broadening due to the Fermi function is $3.5 k_B T$ is between 4.5 μeV and 27.1 μeV. This is much smaller than the smallest superconducting gap (160 μeV), so that in the experiments here, broadening due to the Fermi function does not play a role (although they are part of the fit function). In a normal conducting sample at higher temperature, thermal broadening will eventually become the dominant contribution.

Broadening due to AC modulation. The Josephson curves were measured without AC modulation, while the dI/dV-curves were measured with 10 μeV (peak to peak) AC modulation, which is much smaller than the broadening of the P(E)-function (≈65 μeV). We, therefore, neglect the additional broadening due to AC modulation in the fit model.

RF noise. The contributions from RF noise are difficult to assess. We have specially designed low pass-filters at every feedthrough on the room temperature UHV flanges to block RF noise. In addition, the current and voltage cables going to the STM are high-loss coaxial cables with a distributed RC-filter efficiently blocking high frequency radiation as well. Further, the entire housing of the STM scan head is metallic and at the same base temperature as tip and sample, so that very little (if any) thermal radiation from cryo parts at higher temperature can reach the tunnel junction. We, therefore, believe that the RF noise contribution is small, but ultimately we cannot exclude the possibility that part of the reason why we have an effectively higher fitting temperature is related to RF noise contributions. Assuming that the higher fitted temperature is due to RF noise, we can attribute an effective RF broadening of about 18 μeV.

$P(E)$**-broadening.** A major contribution to the P(E)-broadening is due to the capacitative noise. As the capacitative noise evolves as the square root of the temperature, while the thermal broadening evolves linearly with temperature, capacitative broadening will be dominant at sufficiently low temperatures. Interestingly, while a superconducting tip “eliminates” the effects of thermal broadening, the capacitative broadening remains in any case. In fact, assuming that all electrons are condensed into Cooper pairs, the capacitative broadening for Cooper pairs (CP) is twice as high as for quasiparticles (QP) ($Q_{CP} = 2e$ vs. $Q_{QP} = e$).

SUPPLEMENTARY REFERENCES

[1] Jäck, B. *et al.* A nanoscale gigahertz source realized with josephson scanning tunneling microscopy. *Appl. Phys. Lett.* **106**, 013109 (2015).

[2] Ambegaokar, V. & Baratoff, A. Tunneling between superconductors. *Phys. Rev. Lett.* **10**, 486–489 (1963).

[3] Dynes, R. C., Narayanamurti, V. & Garno, J. P. Direct measurement of Quasiparticle-Lifetime broadening in a Strong-Coupled superconductor. *Phys. Rev. Lett.* **41**, 1509–1512 (1978).
